# Role of Molecular Diagnosis in Imported Cutaneous Leishmaniasis and Its Public Health Significance in India

**DOI:** 10.3390/pathogens14050436

**Published:** 2025-04-30

**Authors:** Rohit Sharma, Deepti Singh, S. Muthukumaravel, S. L. Hoti, Laxmisha Chandrashekar, Manju Rahi

**Affiliations:** 1 Indian Council of Medical Research-Vector Control Research Center, Puducherry 605006, India; rohitpuducherry@gmail.com (R.S.); kumaravelmuthuvel@gmail.com (S.M.); slhoti@yahoo.com (S.L.H.); 2 Department of Dermatology, Jawaharlal Institute of Postgraduate Medical Education and Research (JIPMER), Puducherry 605006, India; deeptisingh2396@gmail.com (D.S.); laxmishac@gmail.com (L.C.)

**Keywords:** *Leishmania*, PCR-RFLP, molecular diagnostics, travel, Liposomal Amphotericin B

## Abstract

Cutaneous leishmaniasis (CL) is a significant public health concern that affects many countries. This disease is caused by the protozoan parasite *Leishmania* spp. and is transmitted through the sandflies from the genus *Phlebotomus* and *Lutzomyia*. The clinical manifestations of CL can vary, often leading to challenges in accurate diagnosis and treatment. In 2022, a 51-year-old male patient presented to a tertiary care hospital in Puducherry, India, with progressively worsening facial lesions and granulomatous plaques. The patient had recently returned from Saudi Arabia, where he likely contracted the infection. Before he visited the tertiary care hospital in Puducherry, the patient had been misdiagnosed and treated for conditions such as Erysipelas and Acute Cutaneous Lupus Erythematosus (ACLE), highlighting the diagnostic challenges associated with CL. Skin scrapings from the patient were subjected to real-time PCR, confirming *Leishmania* spp.’s presence. Cytological examinations revealed the amastigote-like structures within macrophages, thereby establishing the identity of the parasite. For precise species-level identification, PCR-Restriction Fragment Length Polymorphism (PCR-RFLP) and Sanger sequencing of the Internal Transcribed Spacer-1 (ITS-1) region were performed. Molecular techniques confirmed the infection as being caused by *Leishmania tropica*. Following the accurate diagnosis, the patient was successfully treated with Liposomal Amphotericin B, a treatment known for its efficacy against *Leishmania* infections. This case underscores the critical importance of considering cutaneous leishmaniasis in the differential diagnosis of travelers returning from endemic areas who present with dermatological manifestations. The initial misdiagnosis and inappropriate treatment highlight the need for heightened clinical awareness and the utilization of advanced diagnostic tools for accurate identification. Effective and timely treatment, as demonstrated in this case, is essential for the management and control of the disease. This report emphasizes the necessity of vigilance among healthcare providers to recognize and appropriately address imported cases of cutaneous leishmaniasis.

## 1. Introduction

Leishmaniasis is a vector-borne neglected tropical disease caused by *Leishmania* parasites and transmitted by the bite of infected sandflies belonging to the *Phlebetomus* and *Lutzomyia* genus. Leishmaniasis is clinically manifested in three main forms, visceral (VL), mucocutaneous leishmaniasis, and cutaneous (CL) [1]. More than one billion people residing in endemic areas are at risk of leishmaniasis, with 30,000 VL and one million CL new cases reported annually [2]. Approximately 99 countries worldwide are affected by various forms of leishmaniasis. The countries where these infections are most prevalent include Brazil, Ethiopia, India, Kenya, Somalia, South Sudan, and Sudan [2]

Leishmaniasis is still a significant public health problem in India; however, VL cases, a fatal form of leishmaniasis, have declined significantly in the recent past. In 2023, India reported 520 VL cases with four reported deaths. The most recent data indicated 26 cases and zero deaths so far [3]. The clinical management of leishmaniasis mainly relies on treatment with pentavalent antimonials, which are toxic with variable responses in treated patients, and the emergence of drug resistance is leading to relapse of infections [4]. Liposomal amphotericin B is another treatment option for leishmaniasis, but the efficacy of drug depends on the clinical status of the patients [5]. The National Kala Azar Elimination program is aimed to reduce VL incidence to less than one case per 10,000 by the end of 2023 (NCVBDC). The CL infections are restricted to a few parts of north-western India. The Thar desert region of the Rajasthan state and Himachal Pradesh are endemic to CL due to *Leishmania tropica* infection [6]. The recent increase in incidences of atypical CL caused by the *Leishmania donovani* strain, “Zymodeme MON37” in non-endemic areas like Kerala and Himachal Pradesh, is a cause of concern as the strain has been suggested to cause VL in western ghats of southern India [7,8,9]. The southern part of India is not considered as endemic for CL/VL. However, sporadic cases have occurred [7,9,10], and infections in the phlebotomine sandfly fauna have been reported previously [8,11,12,13]. The misdiagnosis of CL for other conditions like Pyoderma Gangrenosum [14], bacterial infections [15], and cutaneous tuberculosis [16] have been reported, especially in areas where CL is not endemic. The current report presents an imported case of CL, and the role of molecular diagnostics in the clinical confirmation of suspected CL cases. This case report also emphasizes the issue of missed diagnoses due to low levels of suspicion among clinicians and the lack of an easy-to-use diagnostic test. This report aims to advocate for the importance of molecular diagnosis in the surveillance of imported CL in the country, which can help prevent potential outbreaks in non-endemic areas.

## 2. Materials and Methods

### 2.1. Case Presentation

**Figure 1:**
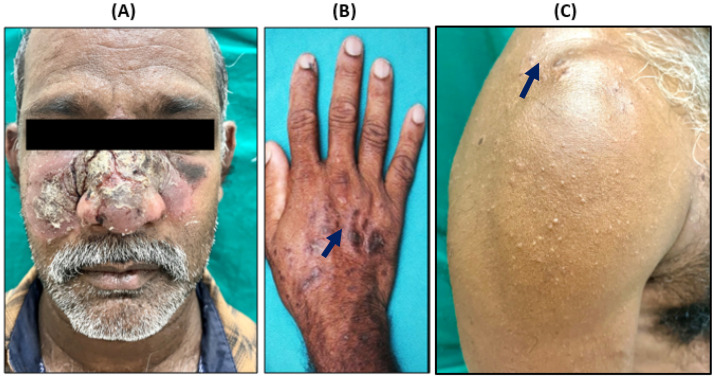
Clinical presentation of reported CL patient. (**A**) Crusted scaly ulcero-necrotic plaque involving center of face with multiple papules, (**B**,**C**) plaque over hand and upper extremities (black arrow).

Cutaneous leishmaniasis (CL) should be suspected in individuals with a history of travel or residence in endemic areas, particularly those with a background of frequent outdoor activities. A 51-year-old male patient working as a laborer in Saudi Arabia, but was a native of the village Konthagai, Tanjore, in Tamil Nadu, India, reported in August 2022 to a tertiary care hospital, situated in Puducherry, southern India. The patient was a migrant laborer working in Saudi Arabia for almost 15 years and had recently returned to his native village in Tamil Nadu, India. He presented with crusted scaly plaques over the center of his face and erythematous papules with central crusting over his upper extremities persisting for almost 3 months when he was in Saudi Arabia (Figure 1A–C). It started as an erythematous scaly crusted plaque over the right malar region and then progressed bilaterally. There was no mucosal involvement. Clinically, classical CL presents as a small, erythematous, painless papule, or plaque primarily on exposed areas, such as the face, arms, and legs, which can gradually progress to develop chronic skin lesions like erythematous nodules, dry scaly crusted plaques, or ulcero-necrotic lesions that resist standard antibiotic and corticosteroid treatment [17]. These lesions may take weeks to months to develop and can result in significant scarring and disfigurement. Atypical presentations include lupoid, eczematous, erysipeloid, zosteriform, paronychia, sporotrichoid, chancriform, and annular forms [18]. Diagnosis can be confirmed through imprint cytology, histopathology, or PCR testing.

### 2.2. Investigations

**Figure 2:**
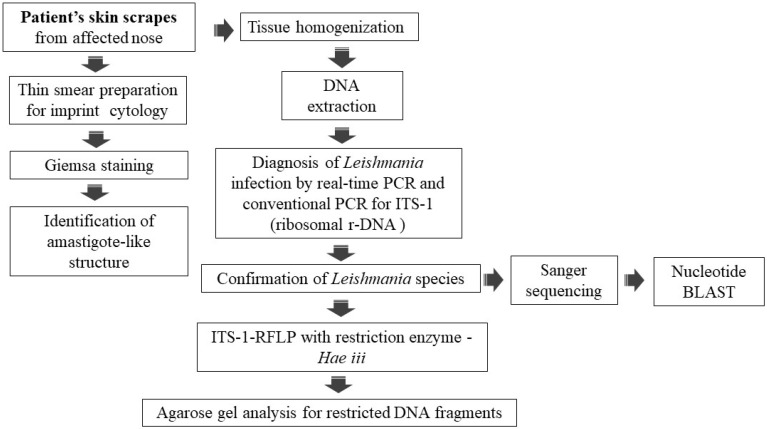
Workflow of laboratory diagnosis of suspected cutaneous leishmaniasis case.

On eliciting the history, it came to be known that before reporting to the tertiary care hospital, the patient was treated empirically with antibiotics and topical steroids in private clinics in India and was misdiagnosed with a case of Acute Cutaneous Lupus Erythematosus (ACLE). At this tertiary care hospital, based on the clinical presentation and history of the patient, CL was suspected. The affected skin scrapes were isolated from the patient for cytological examination, and the molecular diagnosis was performed as per the workflow (Figure 2). The DNA was extracted from skin scrapes using the Qiagen DNeasy blood and tissue extraction kit; briefly the skin scrapes were homogenized in 180 µL of buffer ATL using an automated Tissue Lyser II (Qiagen, Hilden, Germany). The homogenate was processed for DNA extraction using the manufacture’s protocol for tissue extraction. The DNA was eluted in 50 µL of nuclease-free water and used as a template for both TaqMan and conventional PCRs. The PCR amplification for ITS-1 fragment and Hae *III* RFLP was performed as previously described [19]. Briefly, 5 µL of DNA template was used in 25 µL of the PCR reaction containing Dream Taq Master mix (Thermo Scientific, Waltham, MA, USA) and appropriate primers. The RFLP was performed in a 50 µL reaction with 10× buffer, BSA, 1.5 µL of Hae *III* (Promega, Madison, WI, USA), and 10 µL of amplified PCR product incubated at 37 °C for 3 hrs. Restricted PCR products were then analyzed on the 2.5% agarose gel and visualized under UV light. Sanger sequencing of the PCR purified ITS-1 was performed using Big Dye Terminator V3.1 cycle sequencing kit with Genetic Analyzer (Applied Biosystem 3130/3130xl). Sequence alignment was performed with BioEdit software, version 7.2.

### 2.3. Ethics

The signed consent of the patient was obtained before taking the skin samples from the patient. The Institutional Ethical Committee of ICMR-VCRC approved this research study entitled “Role of Molecular Diagnostics in rapid detection of cutaneous leishmaniasis (Approval No: IHEC 02-1024/N/F).

## 3. Results

**Figure 3:**
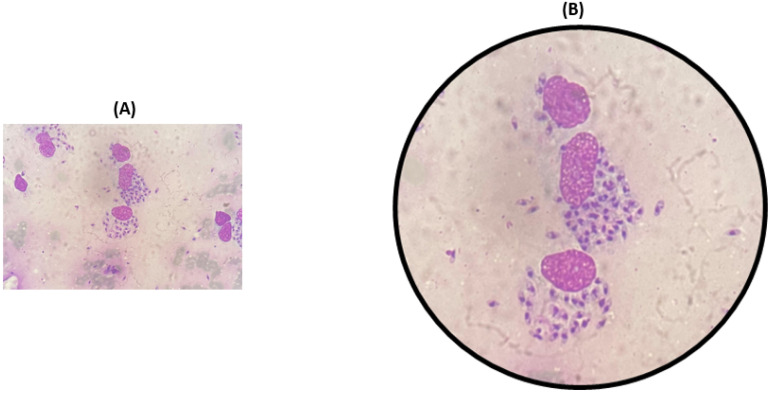
Cytological examinations of thin smear prepared from scrapings of affected nose (**A**), and microscopic view of smear showing intracellular and extracellular amastigotes. (**B**) Macrophages containing amastigote-like forms of leishmania parasites.

The skin scrapes from the affected region, including erythematous plaque over the nose, malar area, and left wrist, showed a similar cellular morphology. Macrophages containing amastigote forms, “*Leishman Donovan*” bodies, were observed in the intracytoplasmic and extracellular space. The cytoplasmic investigation also indicated chronic inflammatory cells like lymphocytes, histocytes, and plasma cells in a hemorrhagic background (Figure 3).

**Figure 4:**
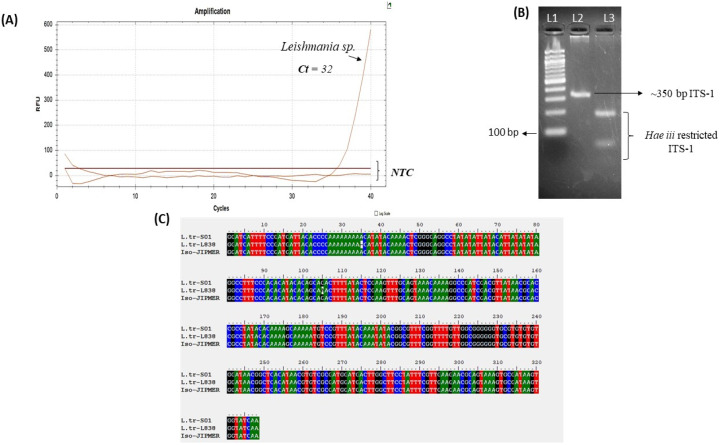
Molecular diagnosis of leishmaniasis case. (**A**) *Leishmania* (genus) specific real-time PCR conducted on affected skin scrapings. (**B**) Restriction fragment length polymorphism (RFLP) of *Leishmania* Internal Transcribed Spacer-1 (ITS-1) amplicon with restriction enzyme *Hae iii*; L1-100 bp marker and L2-350 bp ITS-1 PCR product amplified from DNA of skin scrapings; L3-*Hae iii* digestion of ITS-1 indicating profile similar to *L. tropica.* (**C**) Nucleotide alignment of sequenced *Leishmania* ITS-1 amplicon from skin scrapes (Iso-JIPMER) with *Leishmania* isolate from Malaysia (*L.tr S01-ITS-1*) and Israel (*L.tr-L838-ITS-1*), showing 100% and 99% identity, respectively. NTC-non template control.

Furthermore, the DNA extracted from the skin scrapes was used for a *Leishmania* genus-specific TaqMan-based RT-PCR, which confirmed the presence of *Leishmania* parasites (Figure 4A). To determine the species of parasites ITS-1 amplification and RFLP (PCR-RFLP) with *Hae iii* was performed [19], confirming the presence of the *L. tropica* infection (Figure 4B). Additionally, the PCR amplicon of the ITS-1 fragment was sequenced by the Sanger sequencing method, and the sequence obtained was 100% and 99% identical to the ITS-1 sequences of *L. tropica* patient isolates from Malaysia and Israel, respectively (Figure 4C).

Treatment—The patient was treated with Liposomal Amphotericin B at a dose of 3 mg/kg body weight, on 1, 2, 3, 4, 5, and 10 days.

**Figure 5:**
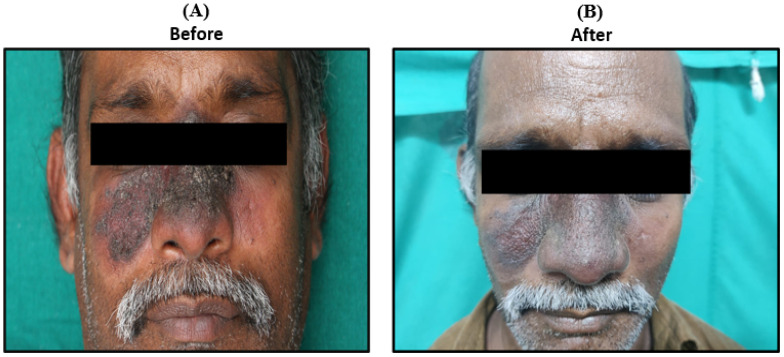
Presentations of clinical symptoms before (**A**) and after 10th day of Liposomal Amphotericin B doses. The patient was treated with 3 mg/kg on days 1, 2, 3, 4, 5, and 10 (**B**). Response on day 10 of treatment (**B**) response after 1-week follow-up.

Outcomes and Follow-up—The patient responded well and showed a significant recovery as documented on day 10 and after one week of follow-up (Figure 5).

## 4. Discussion

Human leishmaniasis is classified as an uncontrolled and emerging infection (class I disease) by the WHO and is responsible for significant mortality and morbidity in over 100 endemic countries [20]. It has a prevalence of 14 million cases, and an estimated population of 350 million are at risk of infection; also, nearly 1.3 million new cases occur each year [1]. The Kingdom of Saudi Arabia (KSA) is one of the most affected countries where CL is a major public health problem [21]. According to the Saudi Ministry of Health, a total of 2763 CL cases were reported between the years 2019 and 2021 [22]. Two important parasites—*Leishmania major*, a zoonotic species, and *L. tropica*, an anthroponotic species—cause CL in KSA, and are transmitted by *Phlebotomus argentipes* and *Phlebotomus sergenti*, respectively [23].

In India, VL, also called Kala-azar, is one of the deadliest parasitic infections in humans and an important public health problem in the country. Significant achievements have been made in India for VL elimination under the ‘Kala-Azar Elimination Programme’. However, occasional outbreaks and a recent increase in incidences of cutaneous leishmaniasis (CL) caused by the *L. donovani* strain, “Zymodeme MON37”, in non-endemic areas like Kerala have been observed, which is a cause for concern because this parasite strain can also cause VL [7,8]. While India has made substantial progress in reducing visceral leishmaniasis (VL) through the Kala-Azar Elimination Programme, emerging CL cases in non-endemic regions, such as Kerala and Himachal Pradesh, indicate a shift in disease dynamics and stress the need for robust surveillance. The presence of susceptible populations and vectors like Phlebotomus species in India raises concerns about the potential for local transmission from imported cases. Furthermore, the new endemic focus of CL caused by *L. tropica* in the state of Himachal Pradesh has been reported, apart from the known endemic area in the Thar region of Rajasthan [6]. Clinical management of leishmaniasis relies mainly on treatment with pentavalent antimonials, which have variable responses in treated patients. Also, these drugs are toxic and the emergence of drug resistance leading to the relapse of infections has been observed [4].

Imported leishmania infections if unreported may facilitate local transmission in non-endemic areas of CL/VL, due to the availability of vulnerable/non-immune human populations and sandfly vectors. These vectors are widely distributed in different geographies of India [24]. The variability in diagnostics and treatment strategies, especially in non-endemic areas of CL, may lead to many undetected imported cases which may contribute to the country’s disease burden. Many Indian migrant workers employed in Middle Eastern countries are at risk of CL/VL. They may import infections with diverse parasite species (*L. tropica*, *Leishmania major*, *Leishmania infantum*, etc.) as there is no surveillance in place at entry points to monitor the infection among travelers coming from endemic areas of leishmaniasis. Recently, imported CL cases caused by *L. major* and several atypical cases of leishmaniasis caused by *L. donovani* have been reported from southern India, which is not endemic for leishmaniasis [9,25]. Useful diagnostic approaches, as presented in Figure 1 and Figure 2, and the awareness of the physicians to seek the travel history in suspected cases of leishmaniasis, or similar presentations like Erysipelas, ACLE, and cutaneous tuberculosis, should be recommended, especially in areas where CL/VL is not prevalent. Molecular approaches that characterize the species of leishmania are critical in understanding the specific clinical presentations and to provide effective treatment.

CL is the most common clinical manifestation of leishmaniasis. *L. tropica* is widely distributed and responsible for many imported cases to non-endemic countries, like Sweden, Turkey, and Japan [26,27,28]. Importantly, *L. tropica* has been recently causing VL in Turkey [29,30] and *L*. *donovani* strains causing both CL and VL are reported in different parts of the world including India. Autochthonous infections of both CL/VL have increasingly been reported in the USA [31]. This has long-term implications for public health in India, as CL-causing Leishmania species can act as a reservoir for VL and contribute to the transmission of VL and potentially change the local disease’s epidemiology. India is slated for VL elimination by 2025 and this scenario can be threatening to the elimination project (less than one case per ten thousand people). It is also critical to identify the visceral strains of *Leishmania* parasites to assess the potential risk mapping and, therefore, molecular surveillance linked with advanced sequencing methods should be highly useful. The genome plasticity of leishmania parasites allows them to withstand drug pressure, leading to the emergence of resistant strains [32]. The plasticity of the genome may also result in a higher diversity of leishmania strains due to genetic recombination which can eventually change the virulence, tropism, and the outcome of the treatment [29,33].

In this report, we present an imported case of CL from a migrant worker returned from Saudi Arabia and confirm the etiology both by cytological and molecular tools. These diagnostics and the characterization by gene sequencing confirmed the infection of *L. tropica* in the nasal scraping sample of the patient. If physicians are sufficiently sensitized by the appropriate health authorities in non-endemic areas to seek the travel history of suspected cases and to confirm the etiology by highly sensitive and specific diagnostics, such as molecular assays of the clinical samples (skin scrapes and punch biopsies) apart from the cytological examination this will ensure correct diagnosis and effective treatment. Molecular diagnostic techniques, particularly Polymerase Chain Reaction-Restriction Fragment Length Polymorphism (PCR-RFLP) and DNA sequencing have revolutionized the identification of *Leishmania* species, offering a precise and reliable approach to diagnosis. PCR-RFLP allows for the detection of specific genetic markers within the *Leishmania* genome, which can differentiate between species with high sensitivity and specificity. Sequencing further enhances accuracy, enabling not only species identification but also the detection of genetic variations, which can inform treatment decisions, especially in areas with emerging resistance. Identifying the correct species of leishmania in these imported cases will also help us understand if the local transmission of CL/VL may be due to a novel species of leishmania, which is yet to be reported in India, i.e., VL by *L. infantum* from a Middle Eastern country. This report emphasizes the need for putting in place a surveillance system for imported disease cases, such as VL, from endemic countries to non-endemic countries and also molecular diagnostics for faster diagnosis.

In conclusion, the integration of molecular diagnostic techniques, such as PCR-RFLP and sequencing, in the clinical management of *leishmaniasis* represents a significant advancement, providing more accurate species identification and contributing to better patient outcomes. Strengthened surveillance, early detection, and targeted treatment strategies, supported by molecular confirmation, are vital to mitigating the risks of imported cases and controlling the spread of leishmaniasis in endemic and non-endemic regions alike. The future establishment of molecular surveillance utilizing clinical samples, like skin scrapes and punch biopsies, of suspected imported leishmaniasis cases can ensure quick and accurate diagnosis for effective management and eventually help mitigate the potential outbreaks in newer areas.

## Data Availability

The original contributions presented in the study are included in the article; further inquiries can be directed to the corresponding author.

## References

[1] Pace D. (2014). Leishmaniasis. J. Infect..

[2] WHO Fact Sheet—Leishmaniasis 2024. https://www.who.int/news-room/fact-sheets/detail/leishmaniasis.

[3] NCVBDC leishmaniasis 2024 Kala-Azar Situation in India: National Center for Vector Borne Diseases Control (NCVBDC). https://ncvbdc.mohfw.gov.in/index4.php?lang=1&level=0&linkid=534&lid=3742.

[4] Sundar S., Chakravarty J., Meena L.P. (2019). Leishmaniasis: Treatment, drug resistance and emerging therapies. Expert Opin. Orphan Drugs.

[5] Frézard F., Aguiar M.M.G., Ferreira L.A.M., Ramos G.S., Santos T.T., Borges G.S.M., Vallejos V.M.R., De Morais H.L.O. (2022). Liposomal Amphotericin B for Treatment of Leishmaniasis: From the Identification of Critical Physicochemical Attributes to the Design of Effective Topical and Oral Formulations. Pharmaceutics.

[6] Sharma N.L., Singh C.D., Sharma R.C., Sharma V.K., Kanga A., Mauricio I., Parwan U.C., Katoch V.M., Mahajan V.K., Sood A. (2005). localized cutaneous leishmaniasis due to *Leishmania donovani* and *Leishmania tropica*: Preliminary findings of the study of 161 new cases from a new endemic focus in himachal pradesh, india. Am. J. Trop. Med. Hyg..

[7] Kumar N.P., Srinivasan R., Anish T.S., Nandakumar G., Jambulingam P. (2015). Cutaneous leishmaniasis caused by *Leishmania donovani* in the tribal population of the Agasthyamala Biosphere Reserve forest, Western Ghats, Kerala, India. J. Med. Microbiol..

[8] Saini P., Kumar N.P., Ajithlal P., Joji A., Rajesh K., Reena K., Kumar A. (2020). Visceral Leishmaniasis Caused by *Leishmania donovani* Zymodeme MON-37, Western Ghats, India. Emerg. Infect. Dis..

[9] Tharakan S.J., Cv D.P., Karthik R., Rupa V., Rose W., Thomas M., Manuel M., Rupali P., Pulimood S., Ajjampur S.S.R. (2021). Case Report: A Single-Center Case Series on Skin Manifestations of Leishmaniasis from a Non-Endemic State in Southern India. Am. J. Trop. Med. Hyg..

[10] Kumaresan M., Kumar P. (2007). Localized cutaneous leishmaniasis in South India: Successful treatment with ketoconazole. Indian J. Dermatol. Venereol. Leprol..

[11] Ilango K., Dhanda V., Srinivasan R., Sadanand A.V., Lane R.P. (1994). *Phlebotomine sandflies* (Diptera: Psychodidae) of Tamil Nadu and Pondicherry, southern India, in relation to visceral leishmaniasis. Ann. Trop. Med. Parasitol..

[12] Srinivasan R., Kumar N.P., Jambulingam P. (2016). Detection of natural infection of *Leishmania donovani* (Kinetoplastida: Trypanosomatidae) in *Phlebotomus argentipes* (Diptera: Psychodidae) from a forest ecosystem in the Western Ghats, India, endemic for cutaneous leishmaniasis. Acta Trop..

[13] Srinivasan R., Jambulingam P., Kumar N.P., Selvakumar M., Edwin B., Kumar T.D. (2015). Temporal distribution and behaviour of sand flies (Diptera: Psychodidae) in a cutaneous leishmaniasis focus of the Kani Tribe settlements in the Western Ghats, India. Acta Trop..

[14] Di Altobrando A., Misciali C., Raone B., Attard L., Gaspari V. (2021). Case Report: Cutaneous Leishmaniasis Misdiagnosed as Pyoderma Gangrenosum. Am. J. Trop. Med. Hyg..

[15] Sikorska K., Gesing M., Olszański R., Roszko-Wysokińska A., Szostakowska B., Van Damme-Ostapowicz K. (2022). Misdiagnosis and inappropriate treatment of cutaneous leishmaniasis: A case report. Trop. Dis. Travel Med. Vaccines.

[16] Cingöz K., Türel Ermertcan A., Temiz P. (2018). Cutaneous leishmaniasis mimicking lupus vulgaris. DOD Clin. Case Rep..

[17] Shrestha A., Mishra A., Mishra A., Shrestha R., Shrestha R. (2024). Uncommon presentation of cutaneous leishmaniasis: Late-onset facial involvement after a decade—A rare case report. Oxf. Med. Case Rep..

[18] Bari A.U., Rahman S.B. (2008). Many faces of cutaneous leishmaniasis. Indian J. Dermatol. Venereol. Leprol..

[19] Mouttaki T., Morales-Yuste M., Merino-Espinosa G., Chiheb S., Fellah H., Martin-Sanchez J., Riyad M. (2014). Molecular diagnosis of cutaneous leishmaniasis and identification of the causative *Leishmania* species in Morocco by using three PCR-based assays. Parasites Vectors.

[20] de Vries H.J.C., Schallig H.D. (2022). Cutaneous Leishmaniasis: A 2022 Updated Narrative Review into Diagnosis and Management Developments. Am. J. Clin. Dermatol..

[21] Abuzaid A.A., Abdoon A.M., Alzahrani A.G., Alhakeem R.F., Asiri A.M., Alzahrani M.H., Memish Z.A. (2017). Cutaneous Leishmaniasis in Saudi Arabia: A Comprehensive Overview. Vector-Borne Zoonotic Dis..

[22] Al-Dhafiri M., Alhajri A., Alwayel Z.A., Alturaiki J.A., Izran S.A.B., Alhammad F.A., Aljumaiah R.M. (2023). Cutaneous Leishmaniasis Prevalence and Clinical Overview: A Single Center Study from Saudi Arabia, Eastern Region, Al-Ahsa. Trop. Med. Infect. Dis..

[23] Haouas N., Amer O., Alshammri F.F., Al-Shammari S., Remadi L., Ashankyty I. (2017). Cutaneous leishmaniasis in northwestern Saudi Arabia: Identification of sand fly fauna and parasites. Parasites Vectors.

[24] Shah H.K., Fathima P.A., Kumar N.P., Kumar A., Saini P. (2023). Faunal richness and checklist of sandflies (Diptera: Psychodidae) in India. Asian Pac. J. Trop. Med..

[25] Fathima P., Shah H.K., Sivalaxmi B., Haritha H., Ajithlal P., Aiswarya R., Saini P. (2024). Genetic diversity of *Leishmania donovani* causing visceral and cutaneous leishmaniasis in the Western Ghats, India. Gene.

[26] Söbirk S.K., Inghammar M., Collin M., Davidsson L. (2018). Imported leishmaniasis in Sweden 1993–2016. Epidemiol. Infect..

[27] Özbel Y., Töz S., Muñoz C., Ortuño M., Jumakanova Z., Pérez-Cutillas P., Maia C., Conceição C., Baneth G., Pereira A. (2022). The current epidemiology of leishmaniasis in Turkey, Azerbaijan and Georgia and implications for disease emergence in European countries. Zoonoses Public Health.

[28] Kitano H., Sanjoba C., Goto Y., Iwamoto K., Kitagawa H., Nomura T., Omori K., Shigemoto N., Hide M., Matsumoto Y. (2021). Complicated cutaneous leishmaniasis caused by an imported case of *Leishmania tropica* in Japan: A case report. Trop. Med. Health.

[29] Özbilgin A., Gencoglan G., Tunali V., Çavuş I., Yıldırım A., Gündüz C., Harman M. (2019). Refugees at the Crossroads of Continents: A Molecular Approach for Cutaneous Leishmaniasis Among Refugees in Turkey. Acta Parasitol..

[30] Alabaz D., Eroğlu F., Elçi H., Çay Ü. (2022). Identification of *Leishmania tropica* from pediatric visceral leishmaniasis in southern mediterranean region of turkey. Mediterr. J. Hematol. Infect. Dis..

[31] Beasley E.A., Mahachi K.G., Petersen C.A. (2022). Possibility of Leishmania Transmission via Lutzomyia spp. Sand Flies Within the USA and Implications for Human and Canine Autochthonous Infection. Curr. Trop. Med. Rep..

[32] Santi A.M.M., Murta S.M.F. (2022). Impact of Genetic Diversity and Genome Plasticity of *Leishmania* spp. in Treatment and the Search for Novel Chemotherapeutic Targets. Front. Cell. Infect. Microbiol..

[33] Glans H., Karlberg M.L., Advani R., Bradley M., Alm E., Andersson B., Downing T. (2021). High genome plasticity and frequent genetic exchange in *Leishmania tropica* isolates from Afghanistan, Iran and Syria. PLoS Neglected Trop. Dis..

